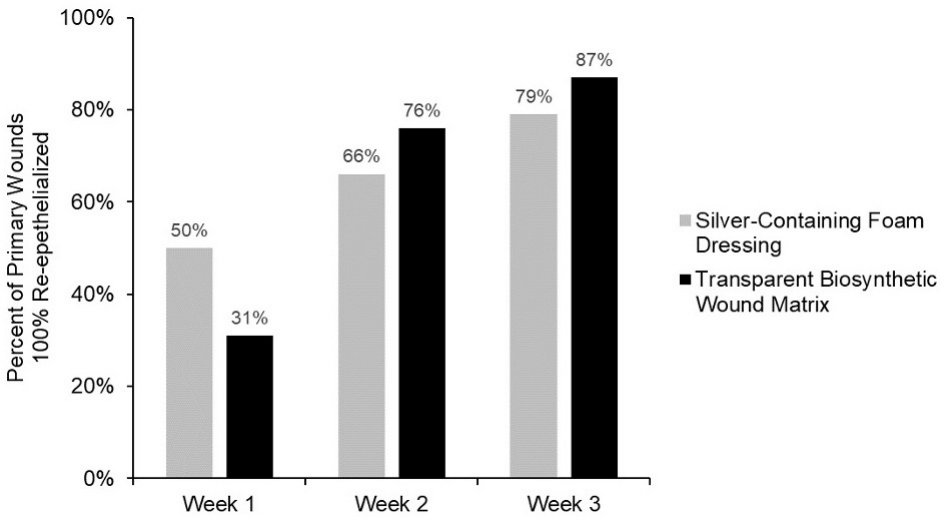# 521 A Randomized Trial Comparing a Transparent Biosynthetic Wound Matrix to Silver-foam Dressing for Partial-thickness Burns

**DOI:** 10.1093/jbcr/iraf019.150

**Published:** 2025-04-01

**Authors:** David Greenhalgh, David Hill, Ram Velamuri, Ian Driscoll, Angela Gibson, Kevin Foster, David Smith, Aleisha Chamberlain, Katie Bush, Miao Yu

**Affiliations:** Shriners Children’s Northern California; Regional One Health; University of South Florida Morsani College of Medicine; University of Florida College of Medicine; University of Wisconsin School of Medicine and Public Health; Diane & Bruce Halle Arizona Burn Center; University of South Florida; AVITA Medical; AVITA Medical; Stedical Scientific

## Abstract

**Introduction:**

A next-generation transparent biosynthetic wound matrix was developed for the treatment of partial-thickness burn injuries. It is composed of an outer slitted 2D silicone epidermal analogue, with customizable pores, which facilitates diffusion of gas and exudate from the wound bed. The silicone sheet is bonded to a nylon knitted fabric coated with porcine gelatin and aloe to induce adherence to the wound until healed. The purpose of this study was to compare the next-generation transparent biosynthetic wound matrix to a commonly used silver-containing foam dressing for the treatment of partial-thickness burns.

**Methods:**

The study was multicenter, randomized, and open-label. Patients with superficial to deep partial-thickness burns < 30% TBSA were included and treated within 72 hours post-injury. Primary outcomes included 100% re-epithelialization (closure) at 2 weeks and time to conversion to alternative therapy. Secondary outcomes included healing at 1 and 3 weeks, adverse events, pain, and number of dressing changes.

**Results:**

A total of 67 patients (42 pediatric & 25 adult) were treated, 32 with the silver foam dressing and 35 with the transparent biosynthetic wound matrix. After 1 week, half the silver foam-treated wounds were closed (p=0.25) (Figure). More patients achieved closure with the transparent biosynthetic wound matrix at weeks 2 and 3 but did not reach statistical significance (p = 0.36 and p = 0.25, respectively) (Figure). Additionally, the transparent biosynthetic wound matrix was associated with significantly lower overall odds of a dressing change (OR 0.15, p=0.67), likely attributable to the adherent nature of the matrix. No differences in conversion to alternate therapies were noted. Overall pain scores and adverse events, including infection, were similar between groups.

**Conclusions:**

The biosynthetic wound matrix is a safe and effective treatment for partial-thickness burns. The biosynthetic wound matrix offers the advantages of being transparent to continuously monitor the wound, being adherent to reduce the need for dressing changes and allowing for bathing after 72 hours.

**Applicability of Research to Practice:**

The biosynthetic wound matrix is a safe and effective option for the treatment of partial-thickness burns in pediatric and adult patients, and may offer clinical advantages to the patient and caregivers over a silver impregnated foam dressing.

**Funding for the Study:**

Study funded by manufacturer